# Characterization of the Vaginal Microbiota among Sexual Risk Behavior Groups of Women with Bacterial Vaginosis

**DOI:** 10.1371/journal.pone.0080254

**Published:** 2013-11-13

**Authors:** Christina A. Muzny, Imran R. Sunesara, Ranjit Kumar, Leandro A. Mena, Michael E. Griswold, David H. Martin, Elliot J. Lefkowitz, Jane R. Schwebke

**Affiliations:** 1 Division of Infectious Diseases, University of Alabama at Birmingham, Birmingham, Alabama, United States of America; 2 Center of Biostatistics and Bioinformatics, University of Mississippi Medical Center, Jackson, Mississippi, United States of America; 3 Biomedical Informatics, Center for Clinical and Translational Sciences, University of Alabama at Birmingham, Birmingham, Alabama, United States of America; 4 Division of Infectious Diseases, University of Mississippi Medical Center, Jackson, Mississippi, United States of America; 5 Division of Infectious Diseases, Louisiana State University Health Sciences Center, New Orleans, Louisiana, United States of America; 6 Department of Microbiology, University of Alabama at Birmingham, Birmingham, Alabama, United States of America; University of Toronto, Canada

## Abstract

**Background:**

The pathogenesis of bacterial vaginosis (BV) remains elusive. BV may be more common among women who have sex with women (WSW). The objective of this study was to use 454 pyrosequencing to investigate the vaginal microbiome of WSW, women who have sex with women and men (WSWM), and women who have sex with men (WSM) with BV to determine if there are differences in organism composition between groups that may inform new hypotheses regarding the pathogenesis of BV.

**Methods:**

Vaginal swab specimens from eligible women with BV at the Mississippi State Department of Health STD Clinic were used. After DNA extraction, 454 pyrosequencing of PCR-amplified 16S rRNA gene sequences was performed. Sequence data was classified using the Ribosomal Database Program classifer. Complete linkage clustering analysis was performed to compare bacterial community composition among samples. Differences in operational taxonomic units with an abundance of ≥2% between risk behavior groups were determined. Alpha and beta diversity were measured using Shannon’s Index implemented in QIIME and Unifrac analysis, respectively.

**Results:**

33 WSW, 35 WSWM, and 44 WSM were included. The vaginal bacterial communities of all women clustered into four taxonomic groups with the dominant taxonomic group in each being *Lactobacillus, Lachnospiraceae*, *Prevotella*, and *Sneathia*. Regarding differences in organism composition between risk behavior groups, the abundance of *Atopobium* (relative ratio (RR)=0.24; 95%CI 0.11-0.54) and *Parvimonas* (RR=0.33; 95%CI 0.11-0.93) were significantly lower in WSW than WSM, the abundance of *Prevotella* was significantly higher in WSW than WSWM (RR=1.77; 95%CI 1.10-2.86), and the abundance of *Atopobium* (RR=0.41; 95%CI 0.18-0.88) was significantly lower in WSWM than WSM. Overall, WSM had the highest diversity of bacterial taxa.

**Conclusion:**

The microbiology of BV among women in different risk behavior groups is heterogeneous. WSM in this study had the highest diversity of bacterial taxa. Additional studies are needed to better understand these differences.

## Introduction

Bacterial vaginosis (BV) is the most common vaginal infection worldwide [1–3]. It is associated with adverse outcomes including preterm birth, low birth weight, postoperative infections, and increased risk of acquisition and transmission of sexually transmitted infections (STIs), including the human immunodeficiency virus (HIV) [4–10]. Microbiologically, BV is characterized by lack of hydrogen peroxide (H_2_O_2_)-producing lactobacilli that characterize the normal vaginal flora and a predominance of facultative (*Gardnerella vaginalis*) and anaerobic (*Prevotella* species (spp.), *Mycoplasma hominis*, *Bacteroides* spp., *Peptostreptococcus* spp., *Fusobacterium* spp., *Mobiluncus* spp., *Atopobium vaginae*, etc.) bacteria [11]. It remains controversial as to whether BV results from acquisition of one organism (i.e. *Gardnerella vaginalis*) as the founder organism leading to the complex microbial community that characterizes BV or whether BV is transmitted by a polymicrobial consortium of micro-organisms [12–14]. BV is most commonly diagnosed using a combination of physical exam and laboratory findings (Amsel criteria) [2] although the diagnosis is more rigorously defined by Gram staining of vaginal fluid to determine the Nugent score [15].

Predictors of BV across studies have included African American race, douching, absence of hormonal contraception, prior history of STIs, and sexual behaviors such as unprotected sex with men, anal sex, and sex with women [2,16–18]. Although BV has never been proven to be a sexually transmitted infection (STI), the epidemiological evidence favoring this is quite robust [11,19–22]. For reasons not well understood, BV may be more common among women who report sex with women (WSW) [23,24]. In the 2001-2004 National Health and Nutrition Examination Survey, BV prevalence was 45.2% among women who reported a history of a female sexual partner compared to 28.8% among women who did not (*p*<0.01) [23]. Others have corroborated this finding, noting the prevalence of BV to range between 22%-52% among WSW [25–32] compared to 23%-40% among women reporting sex with men [33–35]. 

Identification of specific bacterial species present in vaginal fluid can be done using either cultivation-dependent or cultivation-independent molecular methods [12,36–38]. Cultivation-independent molecular methods are increasingly being used because of their ability to detect fastidious micro-organisms that cannot be cultivated by conventional methods. A landmark study by Fredricks et al used a combination of several broad-range PCR techniques to identify bacterial species present in samples of vaginal fluid from women with BV and without BV [38]. In this study, women with BV had a mean of 12.6 bacterial species per sample compared to women without BV who had a mean of 3.3 bacterial species per sample. Nevertheless, despite the use of both cultivation-dependent and cultivation-independent molecular methods, the cause of the complex vaginal flora consistent with BV has remained elusive as Koch’s postulates for establishing disease causation have not been fulfilled for any bacterium or group of bacteria associated with BV [39]. 

Since cultivation-independent molecular methods have become increasingly available, researchers have continued to investigate the vaginal flora of women with and without BV [37,40–43]. However, there is little cultivation-independent data investigating the complex vaginal flora among different sexual risk behavior groups of women with BV. Fethers and colleagues have recently shown with species-specific primers and qPCR that *Megasphaera* type I was 91.3% (95% CI 70.4-98.4) sensitive for BV in WSW but only 72.2% (95% CI 61.2-81.2) sensitive for BV in non-WSW [44]. The authors concluded that BV in WSW has a distinct microbiota compared with other women and acknowledged that additional studies are needed to further explore any potential differences that may be present among these groups of women. The objective of this study was to use 454 pyrosequencing to investigate the vaginal microbiome of WSW, women who have sex with women and men (WSWM), and women who have sex with men (WSM) with BV to determine similarities and differences in organism composition between sexual risk behavior groups that may inform new hypotheses regarding the pathogenesis of this common vaginal infection. 

## Methods

### Study Population and Sample Collection

 This study was approved by the University of Mississippi Medical Center Institutional Review Board (IRB), the Mississippi State Department of Health (MSDH) IRB, and the University of Alabama at Birmingham IRB. All study participants provided written informed consent for use of their samples in research. A cross-sectional selection of stored vaginal swabs taken at a single visit from eligible women with BV aged 18 years or older presenting to the MSDH STD Clinic and enrolled in a Women’s Reproductive Health Program between February 2009 and October 2010 (WSW, WSWM) [45] or a *Mycoplasma genitalium* cohort study between October 2006 and January 2009 (WSM) (data unpublished) were used in this study. All women enrolled in each of these studies were of reproductive age, not pregnant, and reported a history of sexual activity (oral, vaginal, and/or anal) with women only (WSW), with both women and men (WSWM), or with men only (WSM) during the 12 months preceding enrollment. 

 All women had undergone a pelvic examination when enrolled in their respective study. Two vaginal swabs were collected by a study clinician for saline microscopic examination and Gram stain for Nugent score determination [15]. Three out of four Amsel criteria were necessary for the clinical diagnosis of BV and included (1) homogeneous, thin, grayish-white vaginal discharge (2), vaginal pH >4.5 measured by the clinician using pH test strips (EM Science, Gibbstown, NJ) (3), positive whiff-amine test, and (4) clue cells present on a wet mount of vaginal fluid [2]. A Nugent score of 0-3 on vaginal Gram stain was considered indicative of *Lactobacillus*-predominant normal vaginal flora, a score of 4-6 of abnormal, intermediate, vaginal flora, and a score of 7-10 of complex vaginal flora consistent with BV [15]. A third vaginal swab was collected and stored, with participant consent, at -80°C in individual tubes containing 3 mL of phosphate-buffered saline (PBS, pH 7.4) for future research related to the vaginal microbiome. Testing for sexually transmitted infections (STIs) and other vaginal infections was performed for women enrolled in each these studies, as previously described [45] (data unpublished for the *M. genitalium* cohort study). 

 Symptomatic women with BV were treated with metronidazole 500 mg orally twice daily for seven days, as recommended in the 2006 Centers for Disease Control STD Treatment Guidelines [46]. For the purposes of this study, stored vaginal swab specimens collected from women with three or four Amsel criteria and a Nugent score of 7-10 were used for DNA extraction and pyrosequencing. 

### DNA Extraction and Metagenomic Sequencing of Vaginal Microbiota

 DNA extraction was performed on eligible vaginal swab specimens using the QIAamp^®^ DNA mini kit and following the manufacturer’s instructions (QIAGEN, Valencia, CA). Bacterial tag-encoded FLX amplicon pyrosequencing (454 pyrosequencing) was performed by the Research and Testing Laboratories (Lubbock, TX). Briefly, 16S universal Eubacterial primers 28F: 5' - GAGTTTGATCNTGGCTCAG - 3' and 519R: 5' - GTNTTACNGCGGCKGCTG - 3' were used to amplify an approximately 500 base pair genomic locus which included the V1-V3 region of the 16S rRNA gene. Secondary PCR using tagged primers was done before massively parallel pyrosequencing on a Genome Sequencer FLX (Roche, Nutley, NJ). 

### Sequence Data Analysis and Composition

 The QIIME data analysis package was used for 16S rRNA data analysis [47]. Quality filtering on raw sequences (994,670 reads) was performed and sequences which did not fulfill the following criteria were discarded: read length between 200-1000 bp, no ambiguous bases, mean quality score ≥25, and sequences matching the barcode. After quality filtering, a total of 956,793 sequences were used in the final analysis. The number of reads per sample ranged from 2,499 to 25,274 with an average of 8,467 reads per sample. The average read length was 492 bases across all samples; the read length mode was 509 bases. To remove sequence bias specific to pyrosequencing sequences, sequences were “denoised” using the flowgram clustering algorithm present in the QIIME package. 

 Sequences were grouped into operational taxonomic units (OTUs) using the clustering program UCLUST [48] at a similarity threshold of 0.97. In the dataset, 1,480 OTUs were identified in a total of 112 samples. The Ribosomal Database Program (RDP) classifier [49] was used to assign taxonomic category to all OTUs at confidence threshold of 80% (0.8). The RDP classifier uses the 16S rRNA RDP database which has taxonomic categories predicted to the genus level [50]. An OTU table (Table **S1**) was then generated combining all 1,480 OTUs, their taxonomic identification, and their abundance information. Since the RDP classifier provides taxonomic identification of OTUs only to the genus level, an attempt was made to predict species information for several highly abundant OTUs using BLAST (Basic Local Alignment Search Tool) searches against a non-redundant database at the National Center for Biotechnology Information website (available at http://blast.ncbi.nlm.nih.gov). If the lowest common taxonomy for a top blast hit was at the species level, then the species name is mentioned throughout the manuscript. 

 Two tables (Tables **S2** and **S3**) were generated from the OTU table (Table **S1**) for further analysis. For the first table (Table **S2**), the abundance of OTUs present across all samples was normalized to account for different read depth across samples. OTUs whose abundance was not ≥0.1% in at least one of the 112 samples were subsequently filtered. This allowed the removal of “rare taxa” (in the context of sampling depth) [42] leading to a total of 342 most abundant OTUs. Table **S3** was generated based on taxonomy where OTUs belonging to the same taxonomic classification (up to the genus level) were grouped together. This step identified 192 different taxonomic categories from the OTU table. This table was filtered further to select the top 50 most highly abundant taxa across all samples and was then used to generate a heatmap. The heatmap was generated based on hierarchical clustering of Table **S3** using the “heatmap.2” R package, version 2.10.1 (available at http://CRAN.R-project.org/package=gplots). Sexual risk behavior groups, pH (categorized at an interval of 0.5), and Nugent scores were included in the heatmap. Groups of vaginal bacterial communities were identified based on clustering patterns and abundance of taxa. Alpha diversity within the samples was measured using Shannon’s Index implemented in QIIME and beta diversity among the samples was measured using Unifrac analysis [51]. 

### Statistical Analysis

 Statistical analysis was performed using SAS software v9.2 (SAS Institute, Cary, NC). Table **S2** was used to summate similar taxonomic groups. Summary statistics (sum, percentages, mean, standard deviation (SD), minimum, and maximum) were calculated for the overall cohort and for each sexual risk behavior group. Taxonomic groups in Table **S2** with an abundance of ≥2% across the entire sequence library were considered for further analysis. Generalized linear models with negative binomial distributions and log link functions were used to estimate means and relative ratios (RR) of bacterial concentrations for comparing sequence reads between the sexual risk behavior groups. Huber-White robust standard errors and 95% confidence intervals (CIs) were calculated. Alpha was adjusted for multiple-comparison contrasts between sexual risk behavior groups using Bonferroni corrections. A Forest plot [52] was constructed for visual comparison of the relative ratios of bacterial concentrations between sexual risk behavior groups for taxonomic groups with an abundance of ≥ 2% across the entire sequence library. 

## Results

### Description of the Sample

 A total of 112 women with BV (based on Amsel criteria and Nugent score) had stored vaginal specimens that were included in this study: 33 WSW, 35 WSWM, and 44 WSM (Table **1**). Mean age ± standard deviation (SD) of women in each behavioral group was: 25.1 ± 5.0 (WSW), 23.4 ± 4.4 (WSWM), and 23.3 ± 3.8 (WSM). All women in the WSW and WSWM groups were African American (AA); 93.2% of women in the WSM group were AA and 6.8% were Caucasian. The mean vaginal pH ± SD from each risk behavior group was 5.8 ± 0.4 (WSW), 5.6 ± 0.5 (WSWM), and 5.6 ± 0.4 (WSM) and was not statistically significant among sexual risk behavior groups. In addition, there were no significant differences among the Amsel or Nugent scores between groups. 

**Table 1:** Characteristics of sexual risk behavior groups of women with BV (n=112).

Characteristic	WSW (n=33)	WSWM (n=35)	WSM (n=44)
Age (yr)*			
Mean ± SD	25.1 ± 4.9	23.3 ± 4.4	23.2 ± 3.8
Median (Range)	24.5 (21-29)	22.5 (20-27)	23.0 (20-25)
Race			
African American	33 (100%)	35 (100%)	41 (93%)
Caucasian	----	----	3 (7%)
Vaginal fluid pH*			
Mean ± SD	5.7 ± 0.3	5.6 ± 0.5	5.6 ± 0.3
Median (Range)	5.8 (5.5-6.1)	5.5 (5.3-6.1)	5.8 (5.3-5.8)
Amsel score			
Mean ± SD	3.6 ± 0.5	3.6 ± 0.5	3.6 ± 0.5
Median (Range)	4 (3-4)	4 (3-4)	4 (3-4)
Nugent score			
Mean ± SD	9.1 ± 1.1	9.2 ± 1.1	8.9 ± 1.1
Median (IQ Range)	10 (8-10)	10 (8-10)	9 (8-10)

* Two patients have missing data for these variables; n=110

WSW = women who have sex with women; WSWM = women who have sex with women and men; WSM = women who have sex with men; SD = standard deviation; IQ = interquartile range

### Microbiota Distribution in the Sample

A total of 1,480 OTUs were identified from 112 vaginal specimens (Table **S1**). After filtering, 342 highly abundant OTUs were identified (Table **S2**). The most highly abundant OTU across all samples at 26% was OTU-624, identified as bacterial vaginosis-associated bacterium 1 (BVAB1) [38]. This was followed by OTU-1181 (*Lactobacillus iners*) at 11%, OTU-771 (*Snethia*) at 9%, OTU-436 (*Megasphaera*) at 7%, OTU-625 (*Atopobium vaginae*) at 4.5%, and OTU-1377 (*Prevotella bivia*) at 4.3%. OTU-203 and OTU-1230, identified as BVAB2 [38] and BVAB3 [38], had an abundance of 0.7% and 0.1%, respectively. 

**Figure 1:**
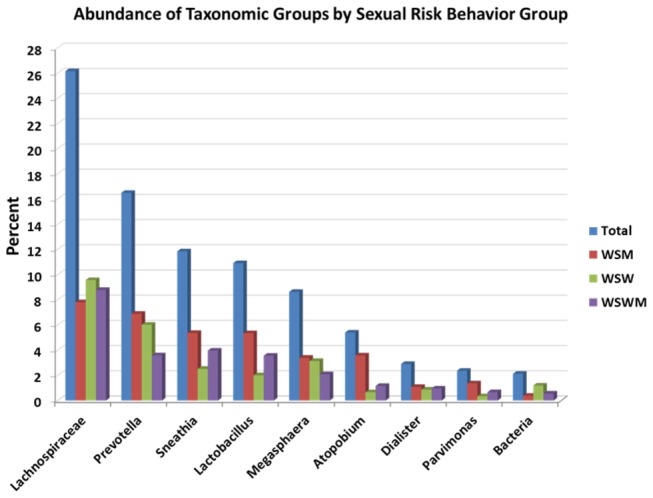
Bar graph of the most abundant OTUs within the total sample and within each sexual risk behavior group. The bacteria group represents sequences which cannot be classified beyond the kingdom (bacteria) level.

OTUs having similar taxonomy were clustered and the top 50 most abundant taxa were identified (Table **S3**). This table was used for taxonomy-based analysis and heatmap generation. The most abundant taxa in the bacterial communities were, in descending order, *Lachnospiraceae* (primarily BVAB1) at 26%, *Prevotella* (primarily *Prevotella bivia* *str*, *P. amnii*, *P. timonensis*, and *P. buccalis*, in descending order) at 16.5%, *Sneathia* at 11.7%, *Lactobacillus* (all *L. iners*) at 11%, *Megasphaera* at 8.4%, *Atopobium* (primarily *Atopobium vaginae*) at 5.4%, *Dialister* at 2.9%, and *Parvimonas* at 2.3% (Figure **1**). 

**Figure 2:**
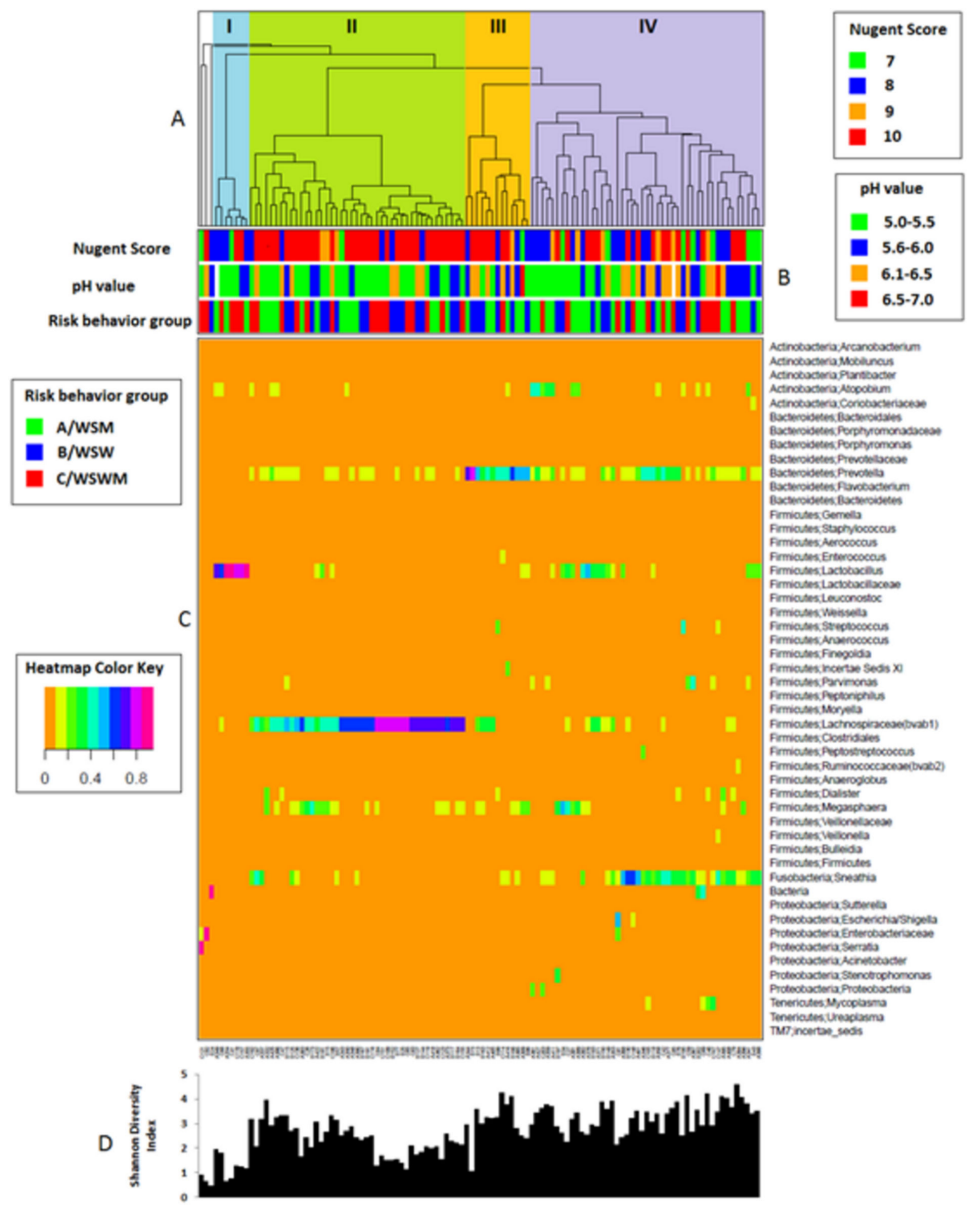
Heatmap with colors showing the abundance of microbial taxa (in proportion) found in the vaginal bacterial communities of 112 women with BV (color key is indicated to the left of the heatmap diagram). (A) Complete linkage clustering of samples based on the species composition and abundance of vaginal bacterial communities that define clusters I-IV. (B) Nugent score, pH measurement, and sexual risk behavior group for each of the 112 vaginal bacterial community samples (color keys for Nugent scores, pH measurements, and sexual risk behavior groups are to the right and the left of the heatmap diagram). (C) Complete linkage clustering of taxa based on Spearman’s correlation coefficient profiles. (D) Shannon diversity indices calculated from the 112 vaginal bacterial communities.

Cluster analysis revealed four major groups of bacterial communities (I – IV) (Figure **2**) with the dominant taxonomic group in each being *Lactobacillus* (I), *Lachnospiraceae* (II), *Prevotella* (III), and *Sneathia* (IV). Three samples were considered as outliers with each having a major proportion of unidentified bacteria, *Enterobacteriaceae*, or *Serratia*. The mean Nugent score ± SD of each cluster was 8.4 ± 1.1 (I), 9.6 ± 0.8 (II), 9.1 ± 1.1 (III), and 8.7 ± 1.1 (IV) (data not shown). 

### Microbiota Distribution in the Sexual Risk Behavior Groups

**Figure 3:**
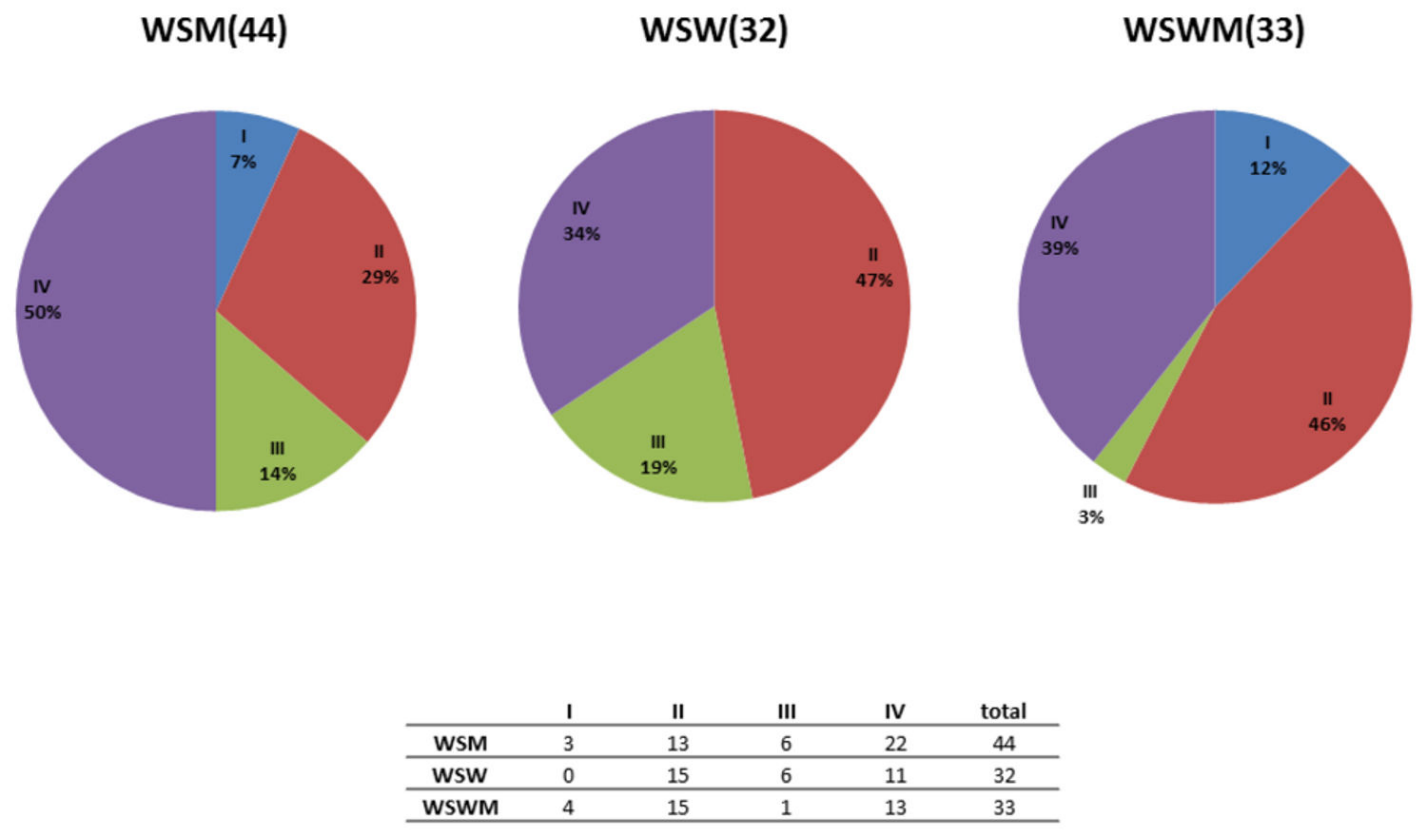
Pie chart representation of the vaginal bacterial communities comprising clusters I-IV within each sexual risk behavior group of women. The number of women from each sexual risk behavior group is indicated in parentheses. Note that 3 samples from the WSWM group contained taxonomic groups that were considered as outliers (i.e., had a major proportion of unidentified bacteria, *Enterobacteriaceae*, or *Serratia*) and were not included in this analysis.

Figures **2** and **3** depict the cluster abundance among the sexual risk behavior groups. Fisher’s exact test was used to determine if there were any significant differences in the overall cluster distribution between the sexual risk behavior groups however no statistically significant differences existed (*p*=0.09). Statistical comparisons between cluster distribution and individual sexual risk behavior groups (i.e. WSW and WSM) were not possible due to the small sample size in each group. 

Figure **1** depicts the most abundant taxonomic groups in the bacterial communities, stratified by sexual risk behavior group. Overall, *Lachnospiraceae*, which primarily includes BVAB1, was the most abundant taxonomic group detected in all sexual risk behavior groups. 

**Figure 4:**
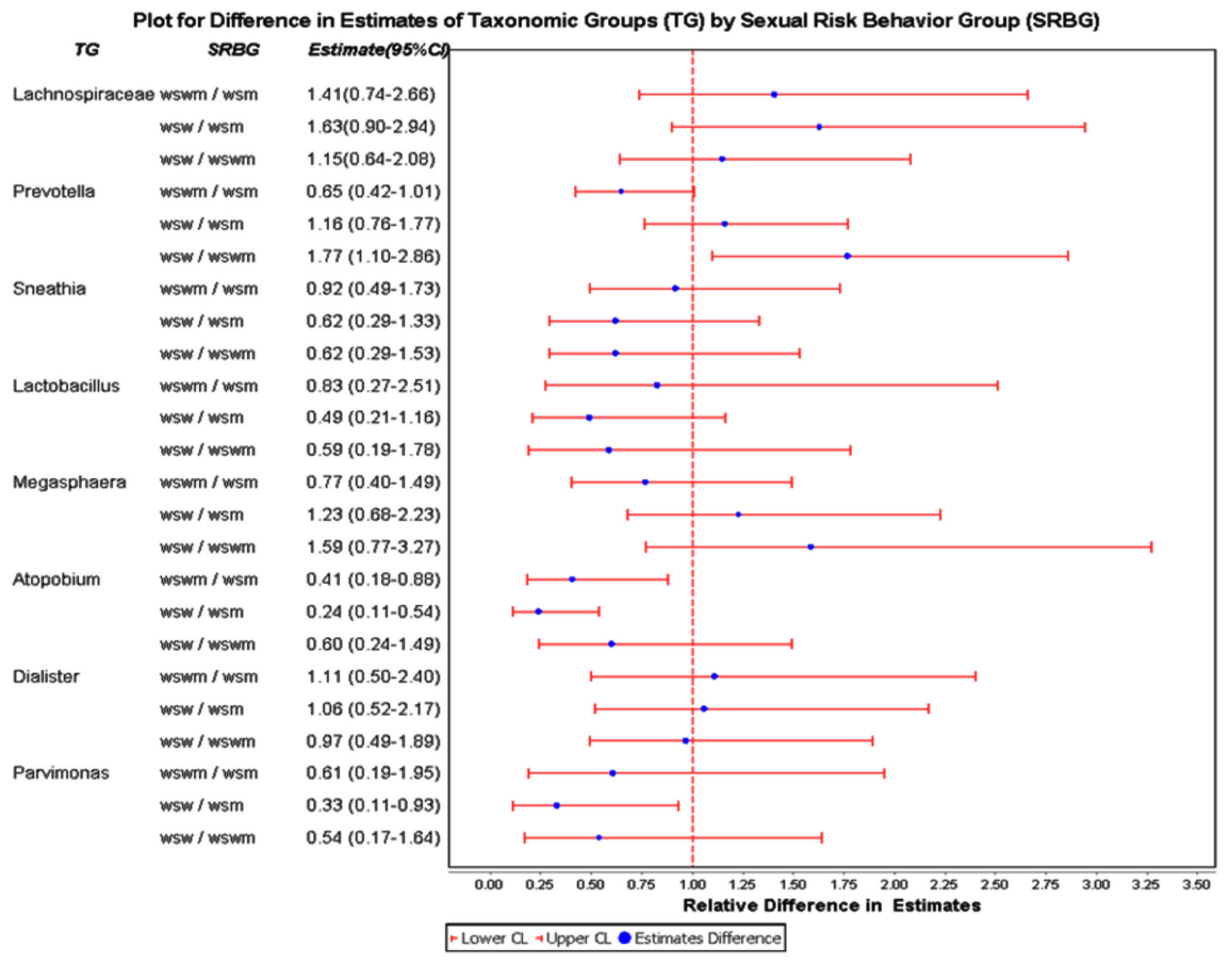
Forest plot depicting the differences in abundance estimates (blue dot) and 95% confidence intervals (CIs) (solid red line) among sexual risk behavior groups of each of the taxonomic groups with an abundance of ≥2% across the entire sequence library. A relative ratio of 1 (dotted vertical line) signifies that both comparison groups have the same mean abundance. If the solid red line for sexual risk behavior group comparisons does not overlap with a relative ratio of 1, a statistically significant difference between the two comparison groups is supported.

Differences in these taxonomic groups between sexual risk behavior groups are represented on a Forest plot (Figure **4**). With regards to comparisons between WSW/WSM groups, the abundance of *Atopobium* (RR=0.24; 95% CI 0.11-0.54) and *Parvimonas* (RR=0.33; 95% CI 0.11-0.93) were significantly lower in the WSW group compared to the WSM group. When comparing WSW/WSWM groups, the abundance of *Prevotella* (RR=1.77; 95% CI 1.10-2.86) was significantly higher in the WSW group than the WSWM group. Finally, when comparing WSWM/WSM groups, the abundance of *Atopobium* (RR=0.41; 95% CI 0.18-0.88) was significantly lower in the WSWM group compared to the WSM group. There were no significant differences with regards to the abundance of *Lachnospiraceae*, *Sneathia*, *Lactobacillus*, *Megasphaera*, or *Dialister* between sexual risk behavior groups. 

**Figure 5:**
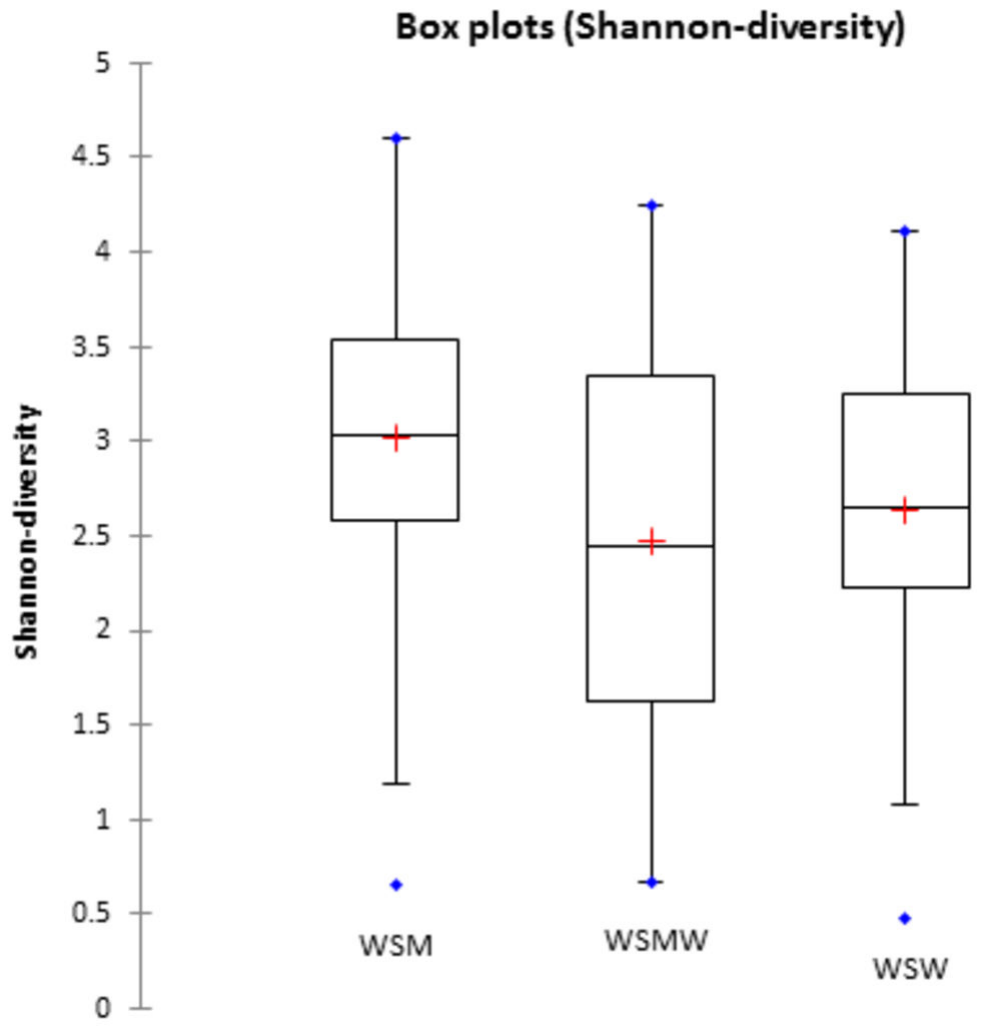
Box plot of Shannon’s diversity for each sexual risk behavior group. The red plus signs depict the average value of Shannon’s diversity for each sexual risk behavior group; blue dots represent outliers. The WSM group had the highest diversity compared to the other sexual risk behavior groups.

**Figure 6:**
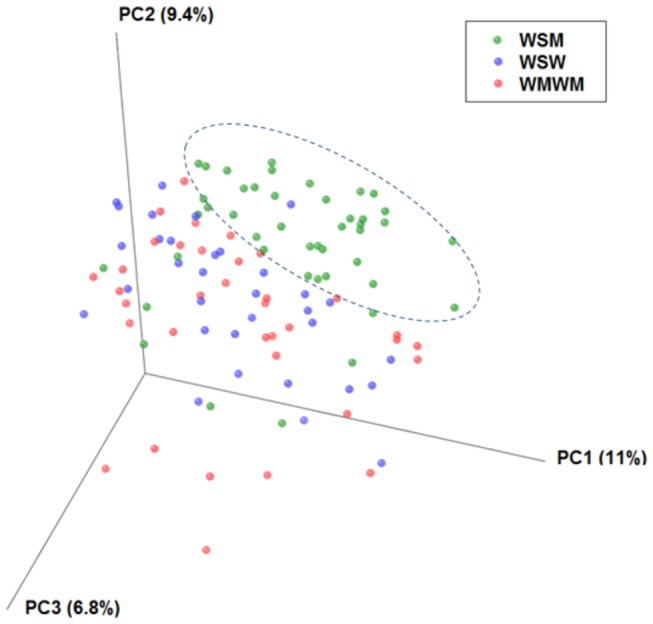
Unweighted beta-diversity Unifrac analysis. This figure depicts a distinct cluster within the WSM group (indicated in green) that is not present within the WSW or WSWM groups (indicated in blue and red, respectively).

 The alpha diversity measurement, depicting diversity within the samples, demonstrated that the WSM group had the highest diversity of bacterial taxa compared to the WSW (*p*=0.04) or WSWM (*p*=0.01) groups (Figure **5**). In addition, the unweighted beta diversity Unifrac analysis (Figure **6**), depicting diversity between the samples (principle components PC1, PC2, and PC3, accounting for the highest percentage of diversity seen, captured 27% variation), showed a distinct cluster within the WSM group. A gtest (which determines whether OTU presence or absence is associated with a category) was performed between the WSM group and the other groups (WSW, WSWM) which revealed that several OTUs were present significantly more often in the WSM group (Bonferonni corrected, *p*<0.05) (data not shown). The WSM group was more likely to have the taxonomic groups *Atopobium* (OTU 519, OTU 313, OTU 19, OTU 492) and *Peptoniphilus* (OTU 1120) and less likely to have the taxonomic group *Plantibacter* (OTU 319) than the WSW and WSWM groups. 

## Discussion

 This study investigated the vaginal microbiome of sexual risk behavior groups of women with BV. *Lachnospiraceae* (primarily BVAB1) was the most abundant taxa across all sexual risk behavior groups (Figure 3). BVAB1, a member of the *Clostridiales* order of anaerobic Gram-positive rods, is a fastidious bacterial taxon which has not yet been cultivated in vitro [38]. Its presence has been found to be highly specific for BV [38] although not all studies have found such a high abundance of BVAB1 among women with BV as our study [43]. It is possible that the abundance of BVAB1 varies based on the characteristics of the population of women with BV studied (i.e. race, ethnicity, sexual practices, etc.); further research is necessary to better understand these differences. In addition, it was interesting to note that, although BV is characterized by a lack of H_2_O_2_-producing lactobacilli, *Lactobacillus* (predominately *L. iners*) was still detectable among women in each of the sexual risk behavior groups in this study. *L iners*, found to be present in both normal and BV microflora [42,53,54] as well as the dominant vaginal bacterium after BV treatment [12,55,56], is hypothesized to facilitate transitions between BV and non-BV states [37,57]. 

 This study found several significant differences in the vaginal flora of predominately African American WSW, WSWM, and WSM with BV. *Atopobium* (primarily *A. vaginae*) was significantly more abundant among WSM than WSWM or WSW (Figures 3, 4). This finding was corroborated by the principal coordinate analysis (Figure 6) which showed a distinct cluster within the WSM group with *Atopobium* being one of the major OTUs in this cluster. It could be hypothesized that *A. vaginae* is not critical to the BV syndrome as WSW (who frequently have a higher prevalence of BV than WSM) have a much lower abundance of *A. vaginae* than WSM, as shown in this study. It may be that *A. vaginae*’s primary niche is in males, that it is sexually transmitted, and that it survives best in the presence of other BV microbiota. In addition, *Parvimonas* was also significantly more abundant among WSM than WSW. *Parviomonas* is a well-known oral pathogen known to be associated with odontogenic infections [58]. Finally, *Prevotella* *spp.* (primarily *P. bivia* *str*, *P. amnii*, *P. timonensis*, and *P. buccalis*), were significantly more common among WSW than WSWM. *Prevotella* *spp.* have been isolated from the oral cavity, the upper respiratory tract, and the urogenital tract in humans [59,60]. *P. bivia* has been noted in women with BV, endometritis, pelvic inflammatory disease, and peri-rectal abscess [61,62] and has been found to produce high lipopolysaccharide concentrations in vaginal fluid which may be an important factor in the pathogenesis of BV and subsequent complications of infection [62]. 

 Taken together, these data seem to suggest that certain Gram-positive anaerobes such as *Atopobium* and *Parvimonas* were significantly more common among WSM with BV than among women reporting sex with women. Additionally, WSM had the highest diversity of bacterial taxa among the sexual risk behavior groups and formed a distinct principal coordinate analysis cluster significantly distant from the other two groups. Potential explanations for these results are not immediately obvious although it could be hypothesized that differential participation in certain types of at-risk sexual practices could be contributing to differences noted in organism composition and BV prevalence among sexual risk behavior groups. Assuming that BV is sexually transmitted, it is currently not well known whether one type of sexual practice (i.e. receptive oral-vulvovaginal sex, receptive oral-anal sex, digital-vaginal sex, digital-anal sex, use of sex toys, etc.) is more important in the pathogenesis of BV than another (i.e. penile-vaginal sex, penile-anal sex, etc.). It is interesting to note that, among WSW participating in a prospective cohort study investigating the risks for BV acquisition, a direct dose-response relationship between increasing numbers of episodes of receptive oral-vulvovaginal sex and BV has been noted [63]. In another cohort of WSW, vaginal insertive use and sharing of sex toys were associated with decreased quantities of vaginal lactobacilli and higher risk of colonization with *G. vaginalis* [64]. Alternatively, studies of primarily heterosexual women have shown an increasing risk of BV in women who have more frequent penile-vaginal sex [19,65]. We did not have detailed sexual practices data (oral, vaginal, and/or anal) with female and/or male sexual partners available for all women in this study and thus cannot make any correlations between BV-associated bacteria and sexual practices at this time. Future studies of the vaginal microbiome among sexual risk behavior groups of women with BV should include this pertinent information. Additionally, it is not well known whether certain BV-associated bacteria prefer the vaginal fluid environment to the male genitourinary environment or whether transmission of infected vaginal fluid is a more efficient mechanism for the acquisition of BV among WSW than transmission from the male genitourinary tract among WSM. Each of these factors could potentially influence differences in BV prevalence noted among sexual risk behavior groups of women. 

 This study has several limitations. First, due to feasibility issues and lack of follow-up in the parent studies from which the WSW, WSWM, and WSM specimens were collected, we did not have paired samples to assess the microbial diversity in the same women pre- and post-resolution of BV. Second, because this was a pilot study with budgetary constraints, we were not able to include an age-matched control group of WSW, WSWM, and WSM with normal vaginal flora to reveal the baseline microbiome in each of these groups of women. Third, the influence of the menstrual cycle and hormone-induced changes in the microbial flora were not able to be controlled for in this study as specimens were obtained from women participating in two different parent studies; future studies in these groups of women should control for phase of the menstrual cycle. Fourth, DNA sequences for amplification of the V1–V3 region of the 16S rRNA gene were generated from a single set of primers which are considered to be conserved among most Eubacteria, however, some taxa such as *Gardnerella* may have divergent sequences which did not efficiently amplify with the primers used here. Previous studies using primers for conserved sequences in 16S rRNA genes have also underrepresented or not consistently amplified 16S rRNA sequences from *Gardnerella* [54,66]. This is an important limitation as *G. vaginalis* has been the bacterium most extensively studied in relationship to BV pathogenesis [14] and is present in 95-100% of clinically diagnosed BV cases using cultivation-dependent methods [34,67,68]. It has recently been detected in the oral and anal cavities of WSW who subsequently developed incident BV, leading to the hypothesis that it may be acquired vaginally from preexisting reservoirs at extravaginal sites [69]. Future studies among sexual risk behavior groups of women with BV should include primers that detect *G. vaginalis* in order to further explore its association with specific sexual practices. Other as yet unknown bacterial taxa associated with BV may have also been missed in this study by using restricted primer sets for DNA amplification. Of note, a recent study by Srinivasan et al which targeted the V3/V4 region of the 16S rRNA gene for broad-range PCR and sequenced the V4 region allowed for high resolution phylogenetic analyses of bacterial communities in women with BV [37]. In addition to not having data on specific sexual practices, we did not have data on recent antibiotic use, hormone use, or vaginal product use which could also have impacted the vaginal microbiomes (with regards to inflammation, pH, and microbial community content) of sexual risk behavior groups of women with BV in this study. Finally, the majority of women in our study were of African American race. Differences in the composition of vaginal microbial communities of women of different racial and ethnic groups with and without BV have been noted [37,42,54] and our results may not be generalizable to other racial and ethnic sexual risk behavior groups of women with BV. Overall, further studies with well-controlled prospective samples, age-matched non-BV controls, and robust questionnaire data are needed to understand whether the differences in vaginal flora identified in this study are valid.

 Despite these limitations, the results of this study demonstrate that the microbiology of BV among women in different sexual risk behavior groups is heterogeneous. How this variation arises is unclear, but it is possible that vaginal, oral, and male urethral secretions are niches for unique communities of micro-organisms which may be transmitted sexually and play important roles in the pathogenesis of BV. Teasing out the specific contributions of sexual practices and sexual partners to the altered vaginal flora of BV should be a priority for future research. Additionally, because treatment of BV is directed at anaerobic organisms, it is critical to define the major anaerobic components of the vaginal flora in BV, as some BV-associated bacteria such as *Atopobium* *spp.* have been shown to be resistant to commonly used agents such as metronidazole [70]. It is possible that different sexual behaviors will define groups of women with BV that respond differently to antibiotic therapy. 

## Supporting Information

Table S1 **Table of 1,480 OTUs present across all 112 vaginal specimens.** This table includes the OTU identification, taxonomy identification, and the abundance (%) in each sample.
(XLS)

Table S2 **Table of the most highly abundant OTUs (in which each OTU comprises ≥0.1% of a sample; n=342) across all 112 vaginal specimens.** This table includes the OTU identification, taxonomy identification, and the abundance (%) in each sample.
(XLS)

Table S3 **Table of the top 50 bacterial taxa (genus level) across all 112 vaginal specimens, based on overall abundance.** This table contains the taxa name (format phylum; genus) and the abundance (%) in each sample.
(XLS)

## References

[1] Fleury FJ (1981) Adult vaginitis. Clin Obstet Gynecol 24: 407-438. doi:10.1097/00003081-198106000-00008. PubMed: 7307366. 7307366

[2] Amsel R, Totten PA, Spiegel CA, Chen KC, Eschenbach D et al. (1983) Nonspecific vaginitis. Diagnostic criteria and microbial and epidemiologic associations. Am J Med 74: 14-22. doi:10.1016/0002-9343(83)91112-9. PubMed: 6600371. 6600371

[3] Rein MF, Holmes KK (1983) Non-specific vaginitis, vulvovaginal candidiasis, and trichomoniasis. In: Remington JS Swartz MN Current Clinical Topics in Infectious Diseases. New York: McGraw-Hill pp. 281-315.

[4] Martin HL, Richardson BA, Nyange PM, Lavreys L, Hillier SL et al. (1999) Vaginal lactobacilli, microbial flora, and risk of human immunodeficiency virus type 1 and sexually transmitted disease acquisition. J Infect Dis 180: 1863-1868. doi:10.1086/315127. PubMed: 10558942. 10558942

[5] Sweet RL (2000) Gynecologic conditions and bacterial vaginosis: implications for the non-pregnant patient. Infect Dis Obstet Gynecol 8: 184-190. doi:10.1155/S1064744900000260. PubMed: 10968604. 10968604 PMC1784684

[6] Leitich H, Bodner-Adler B, Brunbauer M, Kaider A, Egarter C et al. (2003) Bacterial vaginosis as a risk factor for preterm delivery: a meta-analysis. Am J Obstet Gynecol 189: 139-147. doi:10.1016/j.ajog.2003.10.281. PubMed: 12861153. 12861153

[7] Cherpes TL, Meyn LA, Krohn MA, Lurie JG, Hillier SL (2003) Association between acquisition of herpes simplex virus type 2 in women and bacterial vaginosis. Clin Infect Dis 37: 319-325. doi:10.1086/375819. PubMed: 12884154. 12884154

[8] Myer L, Denny L, Telerant R, Souza Md, Wright TC Jr. et al. (2005) Bacterial vaginosis and susceptibility to HIV infection in South African women: a nested case-control study. J Infect Dis 192: 1372-1380. doi:10.1086/462427. PubMed: 16170754. 16170754

[9] Peipert JF, Lapane KL, Allsworth JE, Redding CA, Blume JD et al. (2008) Bacterial vaginosis, race, and sexually transmitted infections: does race modify the association? Sex Transm Dis 35: 363-367. doi:10.1097/OLQ.0b013e31815e4179. PubMed: 18360319. 18360319

[10] Brotman RM, Klebanoff MA, Nansel TR, Yu KF, Andrews WW et al. (2010) Bacterial vaginosis assessed by gram stain and diminished colonization resistance to incident gonococcal, chlamydial, and trichomonal genital infection. J Infect Dis 202: 1907-1915. doi:10.1086/657320. PubMed: 21067371. 21067371 PMC3053135

[11] Hillier SL, Marrazzo J, Holmes KK (2008) Bacterial vaginosis. In: Holmes KK Sparling PF Mardh PA Sexually transmitted diseases. 4th edition. New York: McGraw-Hill pp. 737-768.

[12] Srinivasan S, Fredricks DN (2008) The human vaginal bacterial biota and bacterial vaginosis. Interdiscip Perspect. Infect Dis 2008: 750479. 10.1155/2008/750479 PMC2648628 19282975

[13] Swidsinski A, Doerffel Y, Loening-Baucke V, Swidsinski S, Verstraelen H et al. (2010) Gardnerella biofilm involves females and males and is transmitted sexually. Gynecol Obstet Invest 70: 256-263. doi:10.1159/000314015. PubMed: 21051845. 21051845

[14] Muzny CA, Schwebke JR (2013) *Gardnerella* *vaginalis*: Still a prime suspect in the pathogenesis of bacterial vaginosis. Curr Infect Dis Rep 15: 130-135. doi:10.1007/s11908-013-0318-4. PubMed: 23371405. 23371405

[15] Nugent RP, Krohn MA, Hillier SL (1991) Reliability of diagnosing bacterial vaginosis is improved by a standardized method of gram stain interpretation. J Clin Microbiol 29: 297-301. PubMed: 1706728. 1706728 10.1128/jcm.29.2.297-301.1991 PMC269757

[16] Cherpes TL, Hillier SL, Meyn LA, Busch JL, Krohn MA (2008) A delicate balance: risk factors for acquisition of bacterial vaginosis include sexual activity, absence of hydrogen peroxide-producing lactobacilli, black race, and positive herpes simplex virus type 2 serology. Sex Transm Dis 35: 78-83. doi:10.1097/OLQ.0b013e318156a5d0. PubMed: 17989585. 17989585

[17] Moi H (1990) Prevalence of bacterial vaginosis and its association with genital infections, inflammation, and contraceptive methods in women attending sexually transmitted disease and primary health clinics. Int J STD AIDS 1: 86-94. PubMed: 1965491. 1965491 10.1177/095646249000100203

[18] Barbone F, Austin H, Louv WC, Alexander WJ (1990) A follow-up study of methods of contraception, sexual activity, and rates of trichomoniasis, candidiasis, and bacterial vaginosis. Am J Obstet Gynecol 163: 510-514. doi:10.1016/0002-9378(90)91186-G. PubMed: 2167008. 2167008

[19] Schwebke JR, Desmond R (2005) Risk factors for bacterial vaginosis in women at high risk for sexually transmitted diseases. Sex Transm Dis 32: 654-658. doi:10.1097/01.olq.0000175396.10304.62. PubMed: 16254538. 16254538

[20] Gardner HL, Dukes CD (1955) *Haemophilus* *vaginalis* vaginitis: a newly defined specific infection previously classified non-specific vaginitis. Am J Obstet Gynecol 69: 962-976. PubMed: 14361525. 14361525

[21] Bradshaw CS, Morton AN, Hocking J, Garland SM, Morris MB et al. (2006) High recurrence rates of bacterial vaginosis over the course of 12 months after oral metronidazole therapy and factors associated with recurrence. J Infect Dis 193: 1478-1486. doi:10.1086/503780. PubMed: 16652274. 16652274

[22] Sanchez S, Garcia PJ, Thomas KK, Catlin M, Holmes KK (2004) Intravaginal metronidazole gel versus metronidazole plus nystatin ovules for bacterial vaginosis: a randomized controlled trial. Am J Obstet Gynecol 191: 1898-1906. doi:10.1016/j.ajog.2004.06.089. PubMed: 15592270. 15592270

[23] Koumans EH, Sternberg M, Bruce C, McQuillan G, Kendrick J et al. (2007) The prevalence of bacterial vaginosis in the United States, 2001-2004; associations with symptoms, sexual behaviors, and reproductive health. Sex Transm Dis 34: 864-869. doi:10.1097/OLQ.0b013e318074e565. PubMed: 17621244. 17621244

[24] Evans AL, Scally AJ, Wellard SJ, Wilson JD (2007) Prevalence of bacterial vaginosis in lesbians and heterosexual women in a community setting. Sex Transm Infect 83: 470-475. doi:10.1136/sti.2006.022277. PubMed: 17611235. 17611235 PMC2598706

[25] Marrazzo JM, Koutsky LA, Handsfield HH (2001) Characteristics of female sexually transmitted disease clinic clients who report same-sex behaviour. Int J STD AIDS 12: 41-46. doi:10.1258/0956462011916721. PubMed: 11177481. 11177481

[26] Marrazzo JM, Koutsky LA, Eschenbach DA, Agnew K, Stine K et al. (2002) Characterization of vaginal flora and bacterial vaginosis in women who have sex with women. J Infect Dis 185: 1307-1313. doi:10.1086/339884. PubMed: 12001048. 12001048

[27] Bailey JV, Farquhar C, Owen C, Mangtani P (2004) Sexually transmitted infections in women who have sex with women. Sex Transm Infect 80: 244-246. doi:10.1136/sti.2003.007641. PubMed: 15170014. 15170014 PMC1744826

[28] Pinto VM, Tancredi MV, Tancredi Neto A, Buchalla CM (2005) Sexually transmitted disease/HIV risk behaviour among women who have sex with women. AIDS 19 Suppl 4: S64-S69. doi:10.1097/01.aids.0000192072.80572.43. PubMed: 16249657. 16249657

[29] Evans BA, Kell PD, Bond RA, MacRae KD (1998) Racial origin, sexual lifestyle, and genital infection among women attending a genitourinary medicine clinic in London (1992). Sex Transm Infect 74: 45-49. doi:10.1136/sti.74.1.45. PubMed: 9634303. 9634303 PMC1758086

[30] Edwards A, Thin RN (1990) Sexually transmitted diseases in lesbians. Int J STD AIDS 1: 178-181. PubMed: 2083290. 2083290 10.1177/095646249000100304

[31] McCaffrey M, Varney P, Evans B, Taylor-Robinson D (1999) Bacterial vaginosis in lesbians: evidence for lack of sexual transmission. Int J STD AIDS 10: 305-308. doi:10.1258/0956462991914168. PubMed: 10361919. 10361919

[32] Marrazzo JM, Thomas KK, Agnew K, Ringwood K (2010) Prevalence and risks for bacterial vaginosis in women who have sex with women. Sex Transm Dis 37: 335-339. PubMed: 20429087. 20429087 PMC3291172

[33] Hillier SL, Krohn MA, Rabe LK, Klebanoff SJ, Eschenbach DA (1993) The normal vaginal flora, H_2_O_2_-producing lactobacilli, and bacterial vaginosis in pregnant women. Clin Infect Dis 16 Suppl 4: S273-S281. doi:10.1093/clinids/16.Supplement_4.S273. PubMed: 8324131. 8324131

[34] Eschenbach DA, Hillier S, Critchlow C, Stevens C, DeRouen T et al. (1988) Diagnosis and clinical manifestations of bacterial vaginosis. Am J Obstet Gynecol 158: 819-828. doi:10.1016/0002-9378(88)90078-6. PubMed: 3259075. 3259075

[35] Rifkin SB, Smith MR, Brotman RM, Gindi RM, Erbelding EJ (2009) Hormonal contraception and risk of bacterial vaginosis diagnosis in an observational study of women attending STD clinics in Baltimore, MD. Contraception 80: 63-67. doi:10.1016/j.contraception.2009.01.008. PubMed: 19501217. 19501217

[36] Marrazzo JM, Martin DH, Watts DH, Schulte J, Sobel JD et al. (2010) Bacterial vaginosis: identifying research gaps proceedings of a workshop sponsored by DHHS/NIH/NIAID. Sex Transm Dis 37: 732-744. doi:10.1097/OLQ.0b013e3181fbbc95. PubMed: 21068695. 21068695 PMC3137891

[37] Srinivasan S, Hoffman NG, Morgan MT, Matsen FA, Fiedler TL et al. (2012) Bacterial communities in women with bacterial vaginosis: high resolution phylogenetic analyses reveal relationships of microbiota to clinical criteria. PLOS ONE 7: e37818. doi:10.1371/journal.pone.0037818. PubMed: 22719852. 22719852 PMC3377712

[38] Fredricks DN, Fiedler TL, Marrazzo JM (2005) Molecular identification of bacteria associated with bacterial vaginosis. N Engl J Med 353: 1899-1911. doi:10.1056/NEJMoa043802. PubMed: 16267321. 16267321

[39] Schwebke JR (2009) Bacterial vaginosis: are we coming full circle? J Infect Dis 200: 1633-1635. doi:10.1086/648093. PubMed: 19863440. 19863440

[40] Zozaya-Hinchliffe M, Lillis R, Martin DH, Ferris MJ (2010) Quantitative PCR assessments of bacterial species in women with and without bacterial vaginosis. J Clin Microbiol 48: 1812-1819. doi:10.1128/JCM.00851-09. PubMed: 20305015. 20305015 PMC2863870

[41] Eren AM, Zozaya M, Taylor CM, Dowd SE, Martin DH et al. (2011) Exploring the diversity of *Gardnerella* *vaginalis* in the genitourinary tract microbiota of monogamous couples through subtle nucleotide variation. PLOS ONE 6: e26732. doi:10.1371/journal.pone.0026732. PubMed: 22046340. 22046340 PMC3201972

[42] Ravel J, Gajer P, Abdo Z, Schneider GM, Koenig SS et al. (2011) Vaginal microbiome of reproductive-age women. Proc Natl Acad Sci U_S_A 108 Suppl 1: 4680-4687. doi:10.1073/pnas.1002611107. PubMed: 20534435. 20534435 PMC3063603

[43] Fredricks DN, Fiedler TL, Thomas KK, Oakley BB, Marrazzo JM (2007) Targeted PCR for detection of vaginal bacteria associated with bacterial vaginosis. J Clin Microbiol 45: 3270-3276. doi:10.1128/JCM.01272-07. PubMed: 17687006. 17687006 PMC2045326

[44] Fethers K, Twin J, Fairley CK, Fowkes FJ, Garland SM et al. (2012) Bacterial vaginosis (BV) candidate bacteria: associations with BV and behavioural practices in sexually-experienced and inexperienced women. PLOS ONE 7: e30633. doi:10.1371/journal.pone.0030633. PubMed: 22363457. 22363457 PMC3281856

[45] Muzny CA, Sunesara IR, Martin DH, Mena LA (2011) Sexually transmitted infections and risk behaviors among African American women who have sex with women: does sex with men make a difference? Sex Transm Dis 38: 1118-1125. doi:10.1097/OLQ.0b013e31822e6179. PubMed: 22082722. 22082722

[46] Workowski KA, Berman SM (2006) Sexually transmitted diseases treatment guidelines, 2006. MMWR Recomm Rep 55: 1-94. PubMed: 16888612. 16888612

[47] Caporaso JG, Kuczynski J, Stombaugh J, Bittinger K, Bushman FD et al. (2010) QIIME allows analysis of high-throughput community sequencing data. Nat Methods 7: 335-336. doi:10.1038/nmeth.f.303. PubMed: 20383131. 20383131 PMC3156573

[48] Edgar RC (2010) Search and clustering orders of magnitude faster than BLAST. Bioinformatics 26: 2460-2461. doi:10.1093/bioinformatics/btq461. PubMed: 20709691. 20709691

[49] Wang Q, Garrity GM, Tiedje JM, Cole JR (2007) Naive Bayesian classifier for rapid assignment of rDNA sequences into the new bacterial taxonomy. Appl Environ Microbiol 73: 5261-5267. doi:10.1128/AEM.00062-07. PubMed: 17586664. 17586664 PMC1950982

[50] Cole JR, Wang Q, Cardenas E, Fish J, Chai B et al. (2009) The Ribosomal Database Project: improved alignments and new tools for rRNA analysis. Nucleic Acids Res 37: D141-D145. doi:10.1093/nar/gkp353. PubMed: 19004872. 19004872 PMC2686447

[51] Lozupone C, Hamady M, Knight R (2006) UniFrac - an online tool for comparing microbial community diversity in a phylogenetic context. BMC Bioinformatics 7: 371. doi:10.1186/1471-2105-7-371. PubMed: 16893466. 16893466 PMC1564154

[52] Boyles AL, Harris SF, Rooney AA, Thayer KA (2011) Forest Plot Viewer: a new graphing tool. Epidemiology 22: 746-747. doi:10.1097/EDE.0b013e318225ba48. PubMed: 21811115. 21811115

[53] Falsen E, Pascual C, Sjödén B, Ohlén M, Collins MD (1999) Phenotypic and phylogenetic characterization of a novel *Lactobacillus* species from human sources: description of *Lactobacillus* *iners* sp. nov. Int J Syst Bacteriol 49 1: 217-221. doi:10.1099/00207713-49-1-217. PubMed: 10028266. 10028266

[54] Zhou X, Brown CJ, Abdo Z, Davis CC, Hansmann MA et al. (2007) Differences in the composition of vaginal microbial communities found in healthy Caucasian and black women. ISME J 1: 121-133. doi:10.1038/ismej.2007.12. PubMed: 18043622. 18043622

[55] Ferris MJ, Norori J, Zozaya-Hinchliffe M, Martin DH (2007) Cultivation-independent analysis of changes in bacterial vaginosis flora following metronidazole treatment. J Clin Microbiol 45: 1016-1018. doi:10.1128/JCM.02085-06. PubMed: 17202272. 17202272 PMC1829144

[56] Jakobsson T, Forsum U (2007) *Lactobacillus* *iners*: a marker of changes in the vaginal flora? J Clin Microbiol 45: 3145. doi:10.1128/JCM.00558-07. PubMed: 17652481. 17652481 PMC2045263

[57] Martin DH, Zozaya M, Lillis R, Miller J, Ferris MJ (2012) The microbiota of the human genitourinary tract: trying to see the forest through the trees. Trans Am Clin Climatol Assoc 123: 242-256 PubMed: 23303991. PMC3540603 23303991

[58] Flynn TR, Paster BJ, Stokes LN, Susarla SM, Shanti RM (2012) Molecular methods for diagnosis of odontogenic infections. J Oral Maxillofac Surg 70: 1854-1859. doi:10.1016/j.joms.2011.09.009. PubMed: 22326175. 22326175

[59] Shah HN, Collins DM (1990) *Prevotella*, a new genus to include *Bacteroides* *melaninogenicus* and related species formerly classified in the genus *Bacteroides* . Int J Syst Bacteriol 40: 205-208. doi:10.1099/00207713-40-2-205. PubMed: 2223612. 2223612

[60] Citron DM, Poxton IR, Jo Baron E (2007) Bacteroides, Porphyromonas, Prevotella, Fusobacterium, and Other Anaerobic Gram-Negative Rods. In: Murray PR Jo Baron E Landry ML Manual of Clinical Microbiology, 9th Edition. Washington, DC: ASM Press pp. 911-932.

[61] Jousimies-Somer H (1997) Recently described clinically important anaerobic bacteria: taxonomic aspects and update. Clin Infect Dis 25 Suppl 2: S78-S87. doi:10.1086/516227. PubMed: 9310640. 9310640

[62] Aroutcheva A, Ling Z, Faro S (2008) *Prevotella* *bivia* as a source of lipopolysaccharide in the vagina. Anaerobe 14: 256-260. doi:10.1016/j.anaerobe.2008.08.002. PubMed: 18849004. 18849004 PMC2651005

[63] Marrazzo JM, Thomas KK, Fiedler TL, Ringwood K, Fredricks DN (2010) Risks for acquisition of bacterial vaginosis among women who report sex with women: a cohort study. PLOS ONE 5: e11139. doi:10.1371/journal.pone.0011139. PubMed: 20559445. 20559445 PMC2886123

[64] Mitchell C, Manhart LE, Thomas KK, Agnew K, Marrazzo JM (2011) Effect of sexual activity on vaginal colonization with hydrogen peroxide-producing lactobacilli and *Gardnerella* *vaginalis* . Sex Transm Dis 38: 1137-1144. doi:10.1097/OLQ.0b013e31822e6121. PubMed: 22082725. 22082725 PMC3217189

[65] Bradshaw CS, Morton AN, Garland SM, Morris MB, Moss LM et al. (2005) Higher-risk behavioral practices associated with bacterial vaginosis compared with vaginal candidiasis. Obstet Gynecol 106: 105-114. doi:10.1097/01.AOG.0000163247.78533.7b. PubMed: 15994624. 15994624

[66] Verhelst R, Verstraelen H, Claeys G, Verschraegen G, Delanghe J et al. (2004) Cloning of 16S rRNA genes amplified from normal and disturbed vaginal microflora suggests a strong association between *Atopobium* *vaginae,* *Gardnerella* *vaginalis* and bacterial vaginosis. BMC Microbiol 4: 16. doi:10.1186/1471-2180-4-16. PubMed: 15102329. 15102329 PMC419343

[67] Hill GB (1993) The microbiology of bacterial vaginosis. Am J Obstet Gynecol 169: 450-454. doi:10.1016/0002-9378(93)90339-K. PubMed: 8357043. 8357043

[68] Catlin BW (1992) *Gardnerella* *vaginalis*: characteristics, clinical considerations, and controversies. Clin Microbiol Rev 5: 213-237. PubMed: 1498765. 1498765 10.1128/cmr.5.3.213 PMC358241

[69] Marrazzo JM, Fiedler TL, Srinivasan S, Thomas KK, Liu C et al. (2012) Extravaginal reservoirs of vaginal bacteria as risk factors for incident bacterial vaginosis. J Infect Dis 205: 1580-1588. doi:10.1093/infdis/jis242. PubMed: 22448002. 22448002 PMC3415820

[70] Ferris MJ, Masztal A, Aldridge KE, Fortenberry JD, Fidel PL Jr. et al. (2004) Association of *Atopobium* *vaginae*, a recently described metronidazole resistant anaerobe, with bacterial vaginosis. BMC Infect Dis 4: 5. doi:10.1186/1471-2334-4-5. PubMed: 15018635. 15018635 PMC362875

