# Correction: Characterization of the Vaginal Microbiota among Sexual Risk Behavior Groups of Women with Bacterial Vaginosis

**DOI:** 10.1371/annotation/f7674ab1-fbd5-4293-ad2c-d1a795962e8b

**Published:** 2013-12-03

**Authors:** Christina A. Muzny, Imran R. Sunesara, Ranjit Kumar, Leandro A. Mena, Michael E. Griswold, David H. Martin, Elliot J. Lefkowitz, Jane R. Schwebke

An author was incorrectly omitted from the byline. The name of the author is: Edwin Swiatlo. Prof. Swiatlo is affiliated with institution number 4, and should be placed last in the author order. Prof. Swiatlo's contributions are already correctly indicated in the Author Contributions Statement.

In addition, Prof. Swiatlo should be indicated as the Corresponding Author of the article, and his email address is correct as listed for the author currently indicated as the Corresponding Author.

In addition, there is an error in the affiliation of the eighth author. Jane R. Schwebke is affiliated only with institution number 1, Division of Infectious Diseases, University of Alabama at Birmingham, Birmingham, Alabama, United States of America, and is not affiliated with institution number 4. 

